# Moving MobileMums forward: protocol for a larger randomized controlled trial of an improved physical activity program for women with young children

**DOI:** 10.1186/1471-2458-13-593

**Published:** 2013-06-19

**Authors:** Alison L Marshall, Yvette D Miller, Nicholas Graves, Adrian G Barnett, Brianna S Fjeldsoe

**Affiliations:** 1 Queensland University of Technology, Institute of Health and Biomedical Innovation, School of Public Health and Social Work, Faculty of Health, Brisbane, Australia; 2 The University of Queensland, School of Psychology, Brisbane, Australia; 3 The University of Queensland, School of Population Health, Cancer Prevention Research Centre, Brisbane, Australia

**Keywords:** Text message, SMS, Mobile telephone, Postnatal women, Exercise, Intervention

## Abstract

**Background:**

Women with young children (under 5 years) are a key population group for physical activity intervention. Previous evidence highlights the need for individually tailored programs with flexible delivery mechanisms for this group. Our previous pilot study suggested that an intervention primarily delivered via mobile phone text messaging (MobileMums) increased self-reported physical activity in women with young children. An improved version of the MobileMums program is being compared with a minimal contact control group in a large randomised controlled trial (RCT).

**Methods/design:**

This RCT will evaluate the efficacy, feasibility and acceptability, cost-effectiveness, mediators and moderators of the MobileMums program. Primary (moderate-vigorous physical activity) and secondary (intervention implementation data, health service use costs, intervention costs, health benefits, theoretical constructs) outcomes are assessed at baseline, 3-months (end of intervention) and 9-months (following 6-month no contact: maintenance period).

The intervention commences with a face-to-face session with a behavioural counsellor to initiate rapport and gather information for tailoring the 12-week text message program. During the program participants also have access to a: MobileMums Participant Handbook, MobileMums refrigerator magnet, MobileMums Facebook^©^ group, and a MobileMums website with a searchable, on-line exercise directory. A nominated support person also receives text messages for 12-weeks encouraging them to offer their MobileMum social support for physical activity.

**Discussion:**

Results of this trial will determine the efficacy and cost-effectiveness of the MobileMums program, and the feasibility of delivering it in a community setting. It will inform the broader literature of physical activity interventions for women with young children and determine whether further investment in the translation of the program is warranted.

**Trial registration:**

The trial is registered with the Australian New Zealand Clinical Trials Registry (ACTRN12611000481976).

## Background

Evidence is constantly emerging to support the role of physical activity in the prevention and management of chronic disease [1]. Most developed countries now have public health guidelines for promoting physical activity in adults, yet surveillance data in most countries reveal low guideline compliance [e.g. [2,3]. In Australia, most adults report insufficient levels of physical activity and this guideline deficit is greater in women (62%) than in men (58%) [4]. Australian women with young children (under 5 years old) are less active than women of the same age without children [5] and women with older children [6-8].

Importantly, most women with young children believe in the health benefits that can accumulate from regular physical activity [9-11]. However, previous studies have shown that women with young children lack confidence in being able to include physical activity in their daily lives. Their confidence is eroded by perceived barriers (such as limited access to child care or a lack of instrumental support from their partner) and ideological influences (like their sense of commitment to care for others which leaves them with less time to pursue individual needs) [10-12]. These issues may be overcome by programs that respect women’s multiple roles and provide them with specific cognitive and behavioural skills to overcome barriers and increase their confidence to prioritise physical activity.

Theory-based, individually tailored programs have demonstrated success at increasing physical activity of women with young children [10,13-16]. Previous interventions in this population group have been predominantly delivered by face-to-face contact in either group [13,15,17,18] or individual sessions [16,19]. Although generally effective at increasing physical activity, the evidence from these trials suggests that the requirement for regular face-to-face contact may reduce program attendance [17,20-22]. More recently, researchers have started evaluating broad reach interventions in this population group, using telephone counselling and/or email contact to increase physical activity [23,24]. Emerging research using these mediated (non face-to-face) delivery modes is critical to advancing physical activity interventions for women with young children because it can address issues such as: reaching women from less advantaged backgrounds and across geographic areas; reducing the burden on women accessing programs in structured face-to-face settings; and importantly for public health, potentially reducing the cost of program delivery.

We have spent several years developing MobileMums, a theory-based, tailored physical activity program that responds to the needs of women with young children and is primarily delivered via mobile telephone text messaging [25-27]. In our previous pilot study we found that MobileMums produced short-term (end-of-intervention) increases in the frequency of self-reported moderate-vigorous physical activity [27]. The women in the pilot study were engaged with the program and satisfied that it supported them to increase their physical activity [27]. However, this previous trial: was not adequately powered for examining effects on minutes per week of moderate-vigorous physical activity, did not include a cost-effectiveness analysis, did not include objective measurement of physical activity, and did not assess the longer-term maintenance of the intervention after contact finished. This paper describes the methods of a trial designed to evaluate the efficacy and cost-effectiveness of an improved version of MobileMums (improvements detailed elsewhere [25]) as an intervention to increase the moderate-vigorous physical activity of Australian women with young children. The specific research questions (RQ) being addressed in this randomised controlled trial are:

• RQ1. Does an improved version of MobileMums result in increased levels of moderate-vigorous physical activity?

• RQ2. Is an improved version of MobileMums feasible to deliver and acceptable to participants?

• RQ3. Is MobileMums a cost-effective use of health resources?

• RQ4. What mediated the effect of MobileMums on moderate-vigorous physical activity?

• RQ5. What moderated the effect of MobileMums on moderate-vigorous physical activity?

The results from this trial will provide researchers and community stakeholders with a comprehensive evaluation of the impact of MobileMums and importantly, in the context of limited public health resources and the mediated intervention approach, the potential cost-effectiveness of translating this program into practice.

## Methods

### Study design

MobileMums is being evaluated in a 9-month, two-arm community-based randomised controlled trial. Participants are recruited on a rolling basis and randomly allocated to one of two study groups: the MobileMums intervention group or usual care control group. Data are collected before the program begins (T1), immediately post-intervention (T2, 3 months post baseline), and after a 6-month no contact maintenance period (T3, 9 months post baseline)*.* The final T3 data were collected in December 2012, and the trial is ongoing with further qualitative assessment of program impact. The trial was designed and will be reported in accordance with the CONSORT guidelines for reporting randomised controlled trials [28], and is registered with the Australian New Zealand Clinical Trials Registry (ACTRN12611000481976). Ethical clearance for this research was obtained through the Queensland University of Technology Human Research Ethics Committee.

### Setting

Women with young children were recruited from within a 30 kilometre radius of the Caboolture central business district. Caboolture is located 45 kilometres north of Brisbane, Australia, and had approximately 59,000 residents in 2011 [29]. Caboolture was chosen because it is socio-economically diverse and has a high proportion of women with young children compared with the rest of Australia [29]. This region was also chosen because our research team is involved in an ongoing maternal health partnership with local health service and community organisations. Therefore, if MobileMums is found to be cost-effective, the results from this trial can directly inform the translation of the program within this existing partnership.

### Participant eligibility and recruitment

Women were recruited via one of three methods:

1) An existing database of women with young children who had participated in community surveys about infant and maternal health outcomes in 2006 and had consented to being re-contacted about future research. Each woman was mailed an invitation to participate, which was followed by a personalised text message and telephone call to determine their interest and eligibility.

2) Women were sent an invitation to participate via the Caboolture Early Years Centre Facebook^©^ group. This message was not personally tailored but provided details of the study and asked women to contact research staff via telephone or email.

3) A second database of women with young children who had participated in a survey about maternal health in 2010 and consented to be contacted for further research were mailed an invitation to participate by the Queensland Centre for Mothers & Babies on behalf of the research team. We were not able to contact these women via text message or telephone, so were limited to those who contacted the research staff via telephone, email or reply paid letter in response to the mailed invitation.

Women’s eligibility to participate was assessed via telephone interview. To be eligible, women must: have at least one child aged 5 years or younger; own a mobile telephone; not be pregnant at the time of consent (participants remained eligible if they fell pregnant during the 9-month trial); live within the designated residential area (defined above) and plan to live there for the next 12 months; and, be able to read and understand English. Any woman who had been advised not to exercise by her doctor was first required to receive their doctor’s clearance before participating. Once eligibility was established, women provided informed verbal consent over the telephone.

### Randomisation

In order to achieve similar groups, subjects were randomised in strata according to their baseline physical activity. Baseline physical activity was determined using T1 data from a single item physical activity assessment. The question asks participants to indicate (on a scale from 0–7 days) how many days per week (in the past 3-months) they “exercised for at least 30-minutes”. This single-item question has acceptable criterion validity against Actigraph GT1M accelerometer data (r^s^=0.38, p<0.001) for assessing days per week of at least 30-minutes of moderate-vigorous physical activity in women with young children. We used the data from this brief assessment for stratifying randomisation and not the more detailed T1 physical activity data as the brief assessment is more likely to be used by health agencies in the future to identify potential participants. Each participant was classified as either: not at all active (exercised 0 days per week); somewhat active (exercised between 1 and 4 days per week) or sufficiently active (exercised 5 days or more per week). Randomisation was managed by the project coordinator using lists created by the R software package using random permuted blocks of size ten.

### MobileMums program

MobileMums was developed based on a five step iterative process involving a review of relevant literature and theory, and quantitative and qualitative formative research with the target group [25]. Each component of the MobileMums intervention operationalises at least one construct of the Social Cognitive Theory (self efficacy, goal setting skills, outcome expectancies, social support and perceived environmental opportunity) into a behaviour change technique [25].

Full details of the intervention development process and intricacies of each intervention component are beyond the scope of this manuscript and thus are provided elsewhere [25]. Briefly however, the MobileMums program begins with an initial face-to-face session with a trained MobileMums behavioural counsellor, at which the participants receive a MobileMums Participant Handbook, a MobileMums refrigerator magnet and information brochures, and details for accessing a dedicated MobileMums Facebook^©^ group and a MobileMums website with a searchable, on-line exercise directory. Thereafter, participants receive 12 weeks of tailored theory-based text messages and a follow-up telephone counselling session with their behavioural counsellor at 6 weeks (mid-intervention). Each participant is asked to identify a MobileMums support person. The consenting support person also receives 12 weeks of personalised, theory-based text messages encouraging them to offer instrumental, emotional, or informational support to their MobileMum.

### Initial face-to-face counselling session

The aim of this session is to establish rapport between the participant and their MobileMums counsellor, to collect information to tailor the text messages content, identify a support person, and to initiate the behaviour change process. Participants are guided to: reflect on their previous physical activity patterns; identify expected outcomes of being active; set a SMART physical activity goal and reward for reaching their goal; identify barriers to reaching their goal and strategies to overcome them; and, to identify required support for reaching their goal and a specific person to be their MobileMums support person. To meet the needs of participants this session occurs at a time and location identified by the participant (e.g., their home) and lasts between 25 and 45 minutes.

### MobileMums text messages

Participants receive 52 text messages over the 12-week program: five text messages per week for the first four weeks, and four text messages per week thereafter. Text messages include one ‘goal check message’ sent every Monday. The goal check message asks the participant to respond “yes” or “no” to a message asking whether she met her goal last week or not (e.g., *Jenny did u do all ur planned exercise last wk? Check ur planner magnet & text me back yes or no. Jacqui-MobileMums)*. Once she responds, she is sent a behaviourally-appropriate reply from her MobileMums counsellor (the goal check reply is in addition to the four or five text messages sent each week). Each text message is personalised using the participant’s preferred name and signed off by their MobileMums counsellor’s name. All text messages are tailored to at least one specific Social Cognitive Theory construct (see Table 1) and where appropriate also to the women’s goal, her neighbourhood, her preferred reward or her expected outcomes for reaching her goal. In addition, where appropriate the text messages wording is tailored to the: participant’s youngest child’s name and the support person’s name and gender. The text messages often referred women to other intervention resources such as the Facebook^©^ group, website or handbook.

**Table 1:** Examples of how theoretical constructs are targeted by MobileMums text messages

**Social cognitive theory construct**	**Example text message**
Barrier self efficacy	Jenny take a minute 2 think about how much better u feel after an exercise session. Remember this next time u don’t feel like doing it. Jacqui-MobileMums
Outcome expectancy	Jenny. Don’t feel guilty 4 taking time out 2 exercise, mums say they r more patient & understanding because they exercise. Jacqui -MobileMums
Goal setting skills	Jenny, its OK 2 miss a day now & then, we all do. The trick is 2 get back in2 it ASAP. Review the strategies we planned in ur handbook. Jacqui-MobileMums
Social support	Jenny. Remember Luke wants 2 support u. Make sure he knows what ur MobileMums goal is & what he can do 2 help u meet it. Jacqui-MobileMums
Perceived environmental opportunity	Hi Jenny. MobileMums r enjoying aqua aerobics at Redcliffe Aquatic Centre. Tues & Thurs 4 pm. Costs $6.50. Childcare available. Jacqui-MobileMums

### Support person text messages

The support person is sent three text messages per week. Every second week one of these text messages is tailored to how or whether their MobileMum participant responded to their weekly goal check (e.g., *Luke, congratulate Jenny. She met her goal last wk. Can u help make time 4 her reward? Its a bubble bath. Jacqui- MobileMums*).

### Week 6 telephone counselling session

During Week 6 participants receive a telephone counselling call from their MobileMums counsellor. The aim of this follow-up session is to update the participant’s exercise goals and strategies in order to refine the tailoring content of the text messages sent in Weeks 7–12.

### Other resources

Throughout the program participants have ongoing access to their MobileMums Participant Handbook, MobileMums website with searchable, on-line exercise directory, MobileMums Facebook^©^ group, MobileMums refrigerator magnet, and the state-of-the-art information brochures, all of which they receive at the initial face-to-face consult.

### Usual care control group

Women in the control group receive brief written feedback on their physical activity levels (based on accelerometer data) following each assessment. At baseline, they also receive standard print materials that encourage physical activity. They do not receive any contact with the behavioural counsellor, but do have access to the MobileMums website and a separate, non-moderated Facebook^©^ group that only control participants can access. The treatment of this study group was meant to reflect the standard minimal care that our partner organisations could feasibly deliver without specific funding (e.g., standard print materials and non-moderated Facebook^©^ access), with the exception of accelerometer feedback, which was included to increase participant compliance with assessment procedures and reduce study attrition.

### Data collection procedures

Data are being collected via objective activity monitors (accelerometers), self-administered questionnaire and telephone interview. At each data collection point, participants are sent an assessment package via registered Express Post that contains: an introductory letter; an accelerometer; an accelerometer wear-time logbook; a reply paid registered Express Post mailbag; a self-complete questionnaire; and, an instruction/reference sheet for use during the telephone interview. Two days after the assessment package is sent, the participant is telephoned by research staff to determine if the package has arrived and: 1) provide verbal instruction on how to wear the accelerometer and complete the accelerometer wear-time logbook and questionnaire; and, 2) schedule a time to complete the telephone interview. Three days after the agreed start date for wearing the accelerometer a courtesy phone call is made to ensure correct wear time and placement. A third phone call is made on the expected seventh wear day to prompt efficient return of the accelerometer in the reply paid mailbag. If convenient for the participant, the telephone interview is completed during one of the accelerometer check-in calls; otherwise a separate time convenient to the woman is arranged.

The same data collection procedures are used at both follow-up assessments; however, superfluous socio-demographic variables collected at baseline were removed, and replaced with items to assess participant’s recall, use and satisfaction with the program. All participants receive a nominal gratuity ($20 gift voucher) for each completed assessment to recognise their contribution to the research.

### Primary outcome

The primary outcome is change in moderate-vigorous physical activity (RQ 1). This outcome is being assessed objectively by accelerometer and subjectively through a telephone-administered questionnaire. The accelerometer provides an objective estimate of total accumulated moderate-vigorous physical activity, whereas the self-report questionnaire assesses specific types of moderate-vigorous activity (e.g., brisk walking for exercise). It was important for us to include this self-reported measure of women’s activity, since most women in the pilot study chose to set a SMART exercise goal specific to brisk walking [27], and the accelerometers cannot isolate walking from other moderate intensity activity. Also, MobileMums targets increases in structured, leisure-time physical activity, and the self-report measure allows us to examine activity reported within this domain, while the accelerometer does not differentiate between activity domains.

### Accelerometer

Total accumulated moderate-vigorous physical activity is being assessed using data from Actigraph GT1M accelerometers (Actigraph, LLC, Fort Walton Beach, Florida). Each accelerometer is about the size and shape of a matchbox and is worn on an elastic belt around the waist. It collects data that can be extrapolated into time spent being active (minutes/week) at different intensity levels. Participants are asked to wear it for all waking hours (minimum 10 hours per day) for seven consecutive days, removing it only for sleep or water-based activities. They are asked to record each time they put the accelerometer on or off, as well as any non-wear activities (e.g. water-based activities) in their accelerometer wear-time logbook. The logbook included detailed instructions (with photographs) of how to wear the accelerometer. The accelerometer is set to record data in 1-minute epochs, which will provide output in counts per minute (cpm). Based on a combination of the wear-time logs and the accelerometer data, invalid days of observation (days with < 10 hours wear or excessive counts ≥ 20,000 cpm) will be discarded. Moderate-intensity activity (1952 to 5724 cpm), and vigorous-intensity activity (≥ 5725 cpm) time will be calculated based on standardised cut-points [30]. Data will be reported as averages for valid days and will be summarised to indicate minutes per week of moderate-vigorous activity. The data collection and analytic protocol proposed for this data comply with the best-practice guidelines for conducting accelerometer-based activity assessments in field-based research [31].

### Questionnaire

The Australian Women’s Activity Survey (AWAS) is administered during the telephone interview and was developed to specifically assess physical activity among women with young children [32]. The AWAS assesses women’s typical weekly activity in the past month across five domains (planned, transport, childcare, domestic and work-related) and three intensity levels (light, moderate and vigorous). The interview-administered AWAS has good test-retest reliability (ICC=0.80 (0.65–0.89)) and acceptable criterion validity (compared to accelerometer data; r^s^=0.28, p=0.01) for measuring planned weekly physical activity among women with young children [32]. The key variables that will be extracted from the AWAS are: minutes per week of Planned Moderate-Vigorous Physical Activity; and, minutes per week of Brisk Walking for Exercise. The research staff administering the AWAS received training and conducted role plays before collecting participant data. Throughout the trial, research staff record (with participant consent) two telephone interviews on three separate occasions. A study investigator (BF) listens to these recordings and provides feedback on the AWAS administration in an attempt to increase script fidelity and reduce inter-interviewer variability.

### Secondary outcomes

Secondary outcomes are: program feasibility and participant reports of program acceptability (RQ2); cost-effectiveness (RQ3); potential mediators (RQ4); and, moderators (RQ5).

### Program feasibility

Intervention implementation data are assessed through either participant’s self-report in the paper questionnaire (i.e., treatment of text messages, use of MobileMums refrigerator magnet) or through objective tracking of delivery data (i.e., duration of initial counselling session, number of text messages sent/received, number of goal check text message responses received, number of Facebook^©^ posts, and website usage).

### Program acceptability

Participants’ recall of, use and satisfaction with the program are assessed using self-report items used previously by the investigators [10,27]. Participant responses to the goal check text messages, any unprompted text messages sent by participants, and any additional communication with the behavioural counsellor was monitored [27]. Participants are also asked to describe the MobileMums program in one sentence to provide an unfettered qualitative assessment of the program.

### Cost-effectiveness

Participant-reported use of health care services is assessed in the telephone interview at each data collection point. Participants were asked if they have had any consultations for: their own health with various health professionals; any visits to accident and emergency department; visits by home health nurses; hospital admissions (plus length of stay); and, any other costs associated with taking up any form of physical activity. Costs associated with any reported health service use will be estimated from the Commonwealth Government schedule of re-imbursements [33]. Additional costs associated with program delivery (e.g., computer/database, print materials, text messages, behavioural counsellor time, and other staff costs) were monitored by research staff. Health benefits are collected in the self-administered paper questionnaire at each data collection point using the SF-12v2™ Health Survey [34]. The SF-12 data will be converted to SF-6D utility scores using an established algorithm [35,36]. The SF-6D provides a preference-based value of health benefits derived from standard questions, and a valid estimate of Quality Adjusted Life Years (QALYs) [37]. Change in QALYs will be used as an estimate of health benefit. In line with current theory and recommendations, participant-based production losses will not be included on the cost side of the analysis but (implicitly) counted within the QALY estimation [37,38].

### Theoretical mediators

The MobileMums program is grounded in Social Cognitive Theory and the intervention strategies target change in the specific theoretical constructs proposed [25], therefore the following five constructs are being evaluated as potential mediators via the self-administered questionnaire at each data collection point. *Physical activity barrier self efficacy* is assessed on a 5-point Likert scale (from 1 ‘not at all confident’ to 5 ‘very confident’), using a 12-item tool adapted from a previous scale [39]. Our version includes two additional items for postnatal women (i.e., I could exercise even when: ‘I don’t have anyone to look after the kids’; ‘I have housework to do’). This adapted version of the scale has demonstrated sensitivity to change among postnatal women [10,26] and had acceptable internal consistency (Cronbachs α = 0.71) [26]. *Outcome Expectancy* is measured using 10-item scale developed by Rodgers & Brawley [40]. Participants rate outcome likelihood (on a 10-point scale, 0–100% likelihood) and outcome value (on a 9-point scale, 1–9 importance) for seven physical activity outcomes and ratings are multiplied to indicate overall outcome expectancy (range 0–900). Consistent with the creator’s recommendations, the specific physical activity outcomes used in this study were determined from formative research with the target population. The most commonly reported positive (weight loss, improved fitness, more energy, less stress, improved mental well-being) and negative outcomes (injury, lose time to do other things) were included. Negative outcome expectancies were included because expectations are thought to be better predicted when both positive and negative outcomes are considered [41]. Our previous trial [26] and others [42] have demonstrated that the measure was sensitive to change in a physical activity intervention among postnatal women. The scale has acceptable internal consistency (Cronbachs α = 0.72) [26]. *Goal Setting Skills* are measured using the 10‒item Exercise Goal-setting Scale (EGS)[43]. This scale has good test retest reliability (r = 0.87) over an 8‒week period [43]. The EGS items assesses setting goals (e.g., I often set exercise goals), self-monitoring (e.g., I usually keep track of my progress in meeting my goals) and problem solving (e.g., If I do not reach my goals, I analyse what went wrong). Each item is measured on a 5-point Likert scale, but following our formative research with 12 postnatal women the original anchors (‘does not describe’ to ‘describes completely’) were found to be difficult to interpret so we adapted the EGS anchors to 1 ‘strongly disagree’ to 5 ‘strongly agree’. The adapted version of EGS had good internal consistency (Cronbachs α = 0.84) [26], similar to that of the original scale (r = 0.89)[43]. *Physical activity social support* from the participant’s partner (husband or defacto) and from their family or friends is assessed on a 5-point Likert scale using the Social Support for Exercise Scale [44]. Five items, including an additional one (‘offered to mind the kids so I could be more physically active’) are assessed on a scale from 1 ‘never’ to 5 ‘very often’. The original scale has good test-retest reliability (r = 0.55–0.79)[44], and this slightly modified version has demonstrated good internal consistency (Cronbachs α = 0.90) [26] and sensitivity to change among women with young children [10,26]. At T2 and T3 we included five additional items to specifically assess the support that participants received from their nominated MobileMums support person. *Perceived Environmental Opportunity for Exercise:* was measured using 12 items designed and implemented by Hoehner and colleagues [45]. These items were derived from a review of three commonly used questionnaires to assess environmental impact on physical activity participation [46], and are assessed on a five point Likert scale from 1 ‘strongly disagree’ to 5 ‘strongly agree’. Based on our formative research we added two additional items to the scale (‘There are footpaths wide enough to fit prams in my neighbourhood’; ‘Unattended dogs make it unsafe to walk in my neighbourhood’). The adapted scale has an acceptable internal consistency (Cronbachs α = 0.75) [26].

### Moderators

Demographic (e.g., age, number of children, employment status, education) and health-related (SF12v2™ Health Survey) [34] moderators were assessed in the self-administered questionnaire. Demographic questions follow the same format and response options used in the Census by the Australian Bureau of Statistics [47] to aid interpretation of representativeness of the sample.

### Sample size

Our sample size was based on the clinically meaningful increase in physical activity (assessed by the AWAS) observed in our pilot study (40 minutes/week) [27]. We chose to base the sample size on self-report physical activity data, not the objective accelerometer data, because our pilot data suggested the AWAS was likely to produce the higher sample size estimate due to larger variance. Using the variance observed in the self-report data of 102 minutes/week [27], and assuming 80% power and two-sided significance of 5% we needed 102 women per group. Estimating a 25% dropout, this figure was inflated to 128 per group, or 256 total.

### Data analyses

Data analyses will follow intention-to-treat principles [28], so all participants will be analysed according to their randomised group regardless of whether they complied with the program or not. Missing physical activity data will be imputed using a model that accounts for the often skewed distribution of physical activity. Missing data will be imputed using a regression model based on time and a subject’s previous responses using a random intercept. To account for the uncertainty in imputing missing data the analyses will use multiple imputation using the WinBUGS software. Statistical significance will be set to 5% and 95% confidence intervals will be given for all results.

### Changes in physical activity

Changes in accelerometer-measured moderate-vigorous physical activity (minutes/week) and in AWAS planned moderate-vigorous physical activity and brisk walking (minutes/week) will be analysed using repeated-measures models. Preliminary descriptive analyses will consider the longitudinal trajectories of participants to determine the homogeneity of trajectories and identify outliers. We will fit the repeated measures models using a generalised estimating equations framework. Estimates of the main effects of time by intervention will be used to assess the impact of intervention on each outcome. Possible attrition bias will be assessed using a longitudinal model with a binary dependent variable of dropout at each assessment time. This model will include time-independent covariates such as age, and time-dependent covariates such as previous physical activity.

### Cost-effectiveness analysis

Costs and QALYs will be modelled using a decision analytic Markov model [37], with due consideration of parameter uncertainty as described by probabilistic sensitivity analysis. One thousand random samples will be drawn from probability distributions and cost-effectiveness acceptability curves plotted for the two study groups to reveal the probability the program is cost-effective. This method has been used in previous cost-effectiveness studies [37,48,49].

### Mediator analysis

Potential mediation will be explored using a simple product-of-coefficient approach using Sobel tests [50]. This test will examine whether the ‘indirect effect’ (or the ‘mediated effect’) of the MobileMums intervention is significantly different from zero [50]. The indirect effect is the difference between the ‘total effect’ of the intervention on physical activity and the ‘direct effect’ of the intervention on physical activity after controlling for proposed mediators (Social Cognitive Theory constructs). It should be acknowledged that this trial was not powered to detect mediation and this will be an exploratory analysis.

### Moderator analysis

Exploratory analysis of potential moderators will determine whether intervention effects differ across demographic (e.g., age, gender) and health-related (e.g., BMI, SF-12) characteristics. This analysis will be performed by considering the statistical significance of an interaction between a potential moderator and the intervention as part of the generalised estimating equations model.

## Discussion

This paper describes the methods for evaluating the improved version of the MobileMums program in a large scale community-based randomised controlled trial. The results of this trial will address multiple indicators of program evaluation including efficacy, feasibility, acceptability, cost-effectiveness, mediation and moderation. The results will advance both the science and practice of physical activity interventions for women with young children.

The evidence base for physical activity interventions among women with young children is only starting to include evaluation of mediated (non face-to-face) mechanisms of program delivery [23,24], this is despite the need for highly accessible, flexible program delivery in this population with high caregiving demands. This trial will be the first (other than our pilot evaluation of MobileMums [27]) to evaluate a text message-delivered physical activity intervention for women with young children, and one of the first to evaluate a program delivered primarily by a mediated mechanism for this target group. Results of this trial will also contribute to the evidence for the application of Social Cognitive Theory in interventions to change physical activity and the relative contributions of the theoretical constructs as mediators of change.

Importantly, this trial will provide more valid evidence of the impact of MobileMums on overall physical activity by using objective physical activity measures from an adequately powered sample and assessing maintenance of behaviour change beyond the period of direct program delivery. Assessing the maintenance of behaviour change following an intervention is not common in physical activity trials in general [51] and is very rare within the evidence targeting women with young children. Another strength of this trial is the evaluation of cost-effectiveness of the MobileMums program. Cost reduction for program delivery is one of the key drivers for text message-delivered programs, but there is limited cost-effectiveness evidence to support this rationale.

This thorough evaluation will inform whether further translation of the MobileMums program beyond researcher-administration into community-based practice is warranted. The study investigators are conducting this trial within the context of an existing research-health delivery partnership and thus have the potential to rapidly facilitate the research findings into practice within the target community. We anticipate the findings of this trial will have impact on the practice of physical activity promotion for women with young children within our partnership region. Collectively, the evidence generated by this trial can inform physical activity promotion efforts for women with young children and other populations with accessibility to text messaging interventions, and advance the broader literature for physical activity behaviour change, application of Social Cognitive Theory, and delivery of health behaviour change programs via text messaging.

## Abbreviations

AWAS: Australian women’s activity survey; RQ: Research question.

## Competing interests

The authors declare that they have no competing interests.

## Authors’ contributions

AM conceived the study, led the design of the study, measurement and intervention, coordinated all aspects of study implementation and drafted the manuscript. YM participated in study conception, design and measurement, recruitment of participants, and drafting the manuscript. NG participated in design of the study, measurement, and cost-effectiveness analysis and in drafting the manuscript. AB participated in design of the study, measurement and statistical analyses, conducted the randomisation of participants and participated in drafting the manuscript. BF participated in study conception, recruitment, design and measurement, development of the intervention and drafting of the manuscript, and coordinated the intervention delivery. All authors read and approved the final manuscript.

## Pre-publication history

The pre-publication history for this paper can be accessed here:

http://www.biomedcentral.com/1471-2458/13/593/prepub
